# Synthesis, Characterization and Antiviral Properties of Pd(II) Complexes With Penciclovir

**DOI:** 10.1155/MBD.2001.57

**Published:** 2001

**Authors:** A. Garoufis, K. Karidi, N. Hadjiliadis, S. Kasselouri, J. Kobe, J. Balzarini, E. De Clercq

**Affiliations:** 1 Laboratory of Inorganic and General Chemistry Department of Chemistry University of Ioannina Ioannina 45110 Greece; 2 Department of Chemistry Physics and Material Technology Technological Institution of Athens Egaleo 12210 Greece; 3 National Institute of Chemistry Hajdrihova 19 P.O.B. 3430 Ljubljana Slovenia; 4 Katholieke Universiteit Leuven Rega Institute for Medical Research Minderbroedersstraat 10 Leuven B-3000 Belgium

## Abstract

With the aim to improve and extend the antiviral activity of the antiherpic drug penciclovir, to a wider spectrum of viruses, we have synthesized and characterized new binary and ternary complexes of Pd(II) of formulae *cis*-(pen)_2_PdCl_2_ and *cis*,[(nucl)_2_Pd(pen)_2_]Cl_2_, where nucl = guanosine, inosine, cytidine or penciclovir. The characterization was mainly based on IR and ^1^H NMR spectroscopy, and the results showed that in all prepared complexes, penciclovir coordinates to the metal through N7. The *far*-i.r. spectrum of the complex *cis*-(pen)_2_PdCl_2_ confirmed the *cis*- geometry around Pd(II). All the prepared complexes were markedly active against HSV-1 and HSV-2 strains, but not against thymidine kinase-deficient HSV-1 strains.


					MetalBasedDrugs Vol.8, Nr. 1, 2001
SYNTHESIS, CHARACTERIZATION AND ANTIVIRAL PROPERTIES OF
Pd(II) COMPLEXES WITH PENCICLOVIR
A. Garoufis K. Karidi ,N. Hadjiliadis*l, S. Kasselouri 2 j. Kobe 3
a" J. Balzarini 4
and E. De Clercq4
a
Laboratory ofInorganic and General Chemistry, Department ofChemistry,
University ofloannina, 45110 Ioannina, Greece
b
Department ofChemistry, Physics and Material Technology,
Technological Institution ofAthens, 12210 Egaleo, Greece
National Institute ofChemistry, Hajdrihova 19, P.O.B. 3430, Ljubljana, Slovenia
a Katholieke Universiteit Leuven, Rega Institute for Medical Research,
Minderbroedersstraat 10, B-3000 Leuven, Belgium
ABSTRACT.
With the aim to improve and extend the antiviral activity of the antiherpic drug penciclovir, to a
wider spectrum of viruses, we have synthesized and characterized new binary and ternary complexes of
Pd(II) of formulae cis-(pen)2PdCl2 and cis-[(nucl)2Pd(pen)2]Cl, where nucl guanosine, inosine,
cytidine or penciclovir. The characterization was mainly based on IR and 1H NMR spectroscopy, and
the results showed that in all prepared complexes, penciclovir coordinates to the metal through N7. The
far-i.r, spectrum of the complex cis-(pen)2PdCl2 conf'mned the cis- geometry around Pd(II). All the
prepared complexes were markedly active against HSV-1 and HSV-2 strains, but not against thymidine
kinase-deficient HSV-1 strains.
1. Introduction.
Acyclic nucleoside analogues are well known for their antiviral activity[1]. The antiherpic drug,
acyclovir (ACV), was the first acyclic nucleoside analogue shown to be antivirally effective [2].
Various other guanosine analogues have been synthesized, among which penciclovir or 9(4-hydroxy-3-
(hydroxymethyl)but-l-yl)guanine (Fig. 1). Like acyclovir, penciclovir acts through a selective
inhibition of viral DNA synthesis and replication [3]. For the acyclic nucleoside analogues to be
antivirally active they must be enzymatically metabolized within the herpes virus-infected cells [4].
Thus the interactions of metal ions with acyclic nucleosides and their derivatives present a great
interest, because the majority of enzymes, in virus-infected and uninfected cells, require metal ions for
their activity [5]. Although several metal complexes of acyclovir have been synthesized, characterized
and tested against a variety of viruses [6-10], to our knowledge, until today, there are not reports on the
interaction ofpenciclovir with metal ions.
Herein we report on the synthesis, characterization and antiviral properties of some Pd(II)
complexes with penciclovir.
H2N
O
H
OH
Figure Molecular structure ofPenciclovir
2. Materials and Methods
2.1. Materials andphysical measurements.
The nucleosides guanosine, inosine, cytidine were purchased from Sigma and used without
further purification. Palladium(II) chloride was obtained from Fluka A.G. Penciclovir [11], and the
complexes (guo)2PdCl2, (ino)2PdCl2 and (cyd)2PdCl2 were prepared according to the literature [12].
Mid- and far-i.r, spectra were recorded on a Perkin Elmer GX spectrophotometer in KBr and
polyethylene pellets, respectively. 1H NMR spectra were obtained on a Bruker AMX 400 MHz and on
Bruker AC 250 MHz instrument. The sample temperature was set at 298 K.
2.2 Preparation ofthe complexes. 57
H. Hadjiliadis et al. Synthesis, Characterization andAntiviral
Properties ofPd(II) Complexes with Peniclovir
cis-[Pd(L)2(penh]CI2, cis-[bis(L)bis(penciclovir)palladium(II)]Dichlodde (L guanosine inosine,
cytidine). (I), (II), (IV)
General procedure: cis-[bis(L)dichloropalladium(II)] (L:guo, ino, cyd ) (0.5 mmol) was mixed
with (1 mmol) ofpenciclovir in the solid state, and 2.ml ofD20 was added. After stirring for about 20
min at 50 C complete dissolution was achieved. The 'H-NMR spectrum ofthe mixture showed the end
of the reaction. The complex was purified by chromatographic methods with Sephadex G-10. It was
then precipitated with acetone, filtered off, washed with acetone ( 2 x 5 ml ) and ether ( 2 x 5 ml ) and
f'mally dried at 110 C under vacuum over silica.
(I) [Pd(ino)2(pen)2]Cl2. Anal. Calc. C, 40.3; H, 4.5; N, 21.l.Found: C, 39.9; H, 4.9: N, 20.1.
(II) [Pd(guo)2(pen)2]Cl2. Anal. Calc. C, 39.3; H, 4.6; N, 22.9. Found: C, 38.6; H, 4.9; N, 22.5.
(IV) Pd(cyd)2(pen)2]Cl2. Anal. Calc. C, 39.9; H, 4.9; N, 19.6. Found: C, 39.2; H, 5.2: N, 19.3.
[Pd(pen)]Ci2.] tetrakis(penciclovir)palladium(II) Dichloride. (III)
cis-[bis(penciclovir)dichloropalladium(II)] (0.5 mmol) was mixed with (1 retool) of penciclovir
in the solid state and 2 ml of D20 was added. ARer stirring for about 20 min at 50 C, complete
dissolution was achieved. The evolution of H-NMR spectra showed the end of the reaction. The
complex was then precipitated with acetone, filtered, washed with acetone ( 2 x 5 ml ) and ether ( 2 x 5
ml ), and dried at 110 C under vacuum over silica. The complex was purified by
chromatography on Sephadex G-10
(III) [Pd(pen)4]Cl2. Anal. Calc. C, 42.3; H, 5.3; N, 24.7. Found: C, 41.2; H, 4.9; N, 23.8.
cis-[Pd(pen)Cll, cis-[bis(penciclovir) dichloropalladium(II)]. (V)
Palladium chloride (0.5 mmol) was dissolved in 10 ml of 0.SN HCI by heating to about 50 C.
Penciclovir (1 mmol) was dissolved in 20 ml of 0.SN HCI. The two solutions were mixed at room
temperature and stirred for 2 h. The yellow precipitate that formed was filtered, washed with cold
acetone and ether and dried at 110 C under vacuum.
(V) Pd(pen)2Cl2. Anal. Calc. C, 36.4; H, 4.6; N, 14.6. Found: C, 36.2; H, 4.4; N, 14.8.
3. Results and discussion.
3.1 Synthesis.
The reaction of H2PdCI4 with penciclovir at molar ratio 1:2, in strong acidic solutions (0.SN
HCI), produces the c_is-(pen)2PdCl2, because the trans- influence of pen is comparable to that of
pyridine [13] (eq 1). The ci___ configuration around the metal was confirmed by a Kurnakofftest [14]. A
mixture of the above complex and excess of thiourea, were mixed in the solid state and dissolved in
D20. The HNMR spectrum ofthe mixture showed the presence offree penciclovir.
H2PdC14 + 2 pen '-___cis-(pen)2PdCl2 +2 HCI (1)
The reaction of ci_.s-(pen)2PdCl2 with penciclovir in a molar ratio of 1:2 in aqueous media,
formed the soluble complex [tetrakis(penciclovir)palladium(II)] Dichloride, [Pd(pen)4]Cl2, according
to eq. 2
cis-(pen)2PdCl2 + 2 pen ,,-' [(pen)4Pd]Cl2 (2)
Mixed nucleoside palladium(II) complexes were prepared by a similar procedure upon the
reaction of cis-(guo)2PdCl, ci__s-(ino)2PdCl2 and ci_.s-(cyt)2PdCl2 with free penciclovir.
3.2 Spectroscopic characterization ofthe complexes.
3.2.1. Infraredspectroscopy.
The i.r. spectra ofthe complexes showed a strong band invariably at 1697 to 1726 cm
q
assigned
to free _(C=O) of the 6th
position of the purine ring or the 3t
position of the pyrimidine ring, in the
case of cyd containing complexes, excluding the formation of a Pd-O bond through the carbonyl group
or the coordination through the neighboring N1. A similar behavior was observed in the infrared
spectra of trans- and cis-(nucl)2PdCl or mixed trans- or c__is-[(nucl)2Pd(nucl')2]Cl2, were nucl or nucl_
guanosine, inosine or cytidine [12, 13]. In addition all spectra of the prepared complexes, exhibited a
broad band at about 425 to 430 cmq, attributed to Pd-N stretching vibration, suggesting coordination
through N7. The far-i.r, spectrum of the binary complex ci_s-(pen)PdCl2 exhibited two strong to
medium intensity bands at 325 and 329 cm" assigned to Pd-CI stretching vibration, confirming the cis__=
configuration ofthe chlorine atoms [16].
The characteristic i.r. bands for the complexes and the
a
TABLE I Characteristic r and far-i r bands cm for the complexes and the free ligand
Compounds va(C=O) 15(NH2) v(C=C, C=N)b
v (Pd-N) v (Pd-CI)
Penciclovir 1692 s 1645 m 1604 s 1546 m
ci_s-(pen)2PdC12 1726s 1642s 1625m 1550b 430m 419w 325s 339m
[(pen)4Pd]Cl2 1712 s 1640 s 1620-1530 b 425 w
cis-[(guo)2Pd(pen)2]Cl2 1697 s 1637 s 1598 s 430 w
cis-[(ino)2Pd(pen)2]Cl2 1701 s 1635 s 1592 b 428 w
cis-[(cd)Pd(pe,n)2 (2121720 s 1678 s 1664 s 1620 w 1592 b 430 m
'r-i-:-Sf'rm spectra, skeletal vibrations. Abbreviatins b =::br0ad-m:du :=:strongl
weak.
58
Metal BasedDrugs Vol.8, Nr. 1, 2001
3.2.2 HNMR spectroscopy.
The H8 resonance of penciclovir, in the H NMR spectra of all five prepared complexes showed a
downfield shitt by 0.38 to 0.46 ppm, compared to the free ligand in D20, indicating a covalent interaction of
the Pd(II) ion with the neighboring to HS, nitrogen atom of the purine ring (N7). Similar strong downfield
shifts of the guanosine's H8 proton, were observed in the binary or ternary complexes of the ligand with
Pd(II) [12,13]. It is noticeable that in the spectrum of cis-(pen)2PdClz ( in DCI IN) the H8 proton resonance
shifts upfield, compared with the protonated at N7 form ofpenciclovir, pen (in DCI IN), by about 0.47 ppm,
indicating that the IT causes higher electron deshielding in the magnetic environment ofthe H8 nucleus than
the palladium ion.
TABLE II gives the HNMR chemical shifts ofthe prepared complexes.
TABLE II. 400 MHz HNMR chemical shifts (ppm) b of the prepared complexes and the free ligand.
PENCICLOVIR PROTONS NUCLEOSIDE PROTONS
Compounds solvent H8 HI' H2' H3' H4' & H4" H8 H2 H5
Pencielovir D20 7.90 3.99 1.70 1.45 3.40
DCI 1N 8.69 4.06 1.67 1.46 3.49
cis-(pen)2PdCl2 DCI 1N 8.22 4.02 1.67 1.45 3.52
[(pen)4Pd]Cl2 D20 8.36 4.01 1.69 1.45 3.42
c.is-[(guo)2Pd(pen)2]Cl2 D20 8.39 4.09 1.70 1.39 3.45
e.cj_i[(ino)2Pd(pen)2]Cl2 DzO 8.28 4.06 1.72 1.45 n.a.
8.94
8.98 8.20
cis-[(C,d)2Pd(pen)2]Cl2 D20 8.23 4.05 1.71 1.36 n.a. 8.18.
Spectra recorded at ambient temperature, b
The values are referenced to theHDO peak which has been set at 4.82
ppm.
250 MHz spectra, n.a not assigned.
3.3 Antiviral properties.
All prepared penciclovir complexes were markedly active against HSV-1 and HSV-2 strains but not
against thymidine kinase-defieient (TK') HSV in E6SM cell cultures (TABLE III). The compounds were
also inactive against a variety of other viruses including vesicular stomatitis virus, Coxsackie virus B4 and
respiratory syncytial virus in HeLa cell cultures (TABLE IV) and against parainfluenza-3 virus, reovirus-1,
Sindbis virus, Coxsackie virus B4 and Punta Toro virus in Vero cell cultures (TABLE V). They were also not
active against human immunodeficieney virus type (IIIa) and type 2 (ROD) in CEM and MT-4 cell cultures
(data not shown). None of the compounds proved markedly cytostatic against murine leukemia L1210,
murine mammary carcinoma FM3A and human lymphocyte Molt4 and CEM cells (50% inhibitory
concentration > 100 tM) (except for compound II that inhibited Molt4 and CEM cell proliferation at 30-34
tM) (TABLE VI). Clearly, the compounds I-V displayed a similar antiviral spectrum as the parent
compound peneiclovir. However, they were not superior to penciclovir in inhibiting herpes virus-induced
cytopathicity in cell culture. Also, the test compounds lost marked activity against a TK-defieient herpes
simplex virus as also penciclovir did. In general, the most active compound was III that contained four
pencielovir molecules for each Pd atom in the entire molecule.
In conclusion, the Pd containing penciclovir derivatives had a comparable antiviral specmun as
penciclovir (i.e. herpes simplex virus type 1 and 2), but were not superior to the parent compound.
3.4 Broad-spectrum antiviral activity assays
For herpes simplex viruses (HSV), vaccinia virus (VV), Coxsackie virus type B4, vesicular
stomatitis virus (VSV), parainfluenza virus type 3, respiratory syneytial virus (RSV), Sindbis virus, Punta
Toro virus and reovirus type 1, the origin ofthe virus stocks [17] and the assay procedures [18, 19] have been
described previously. HSV assays were carried out against HSV-1 TK+
(KOS, F and Mclntyre) and HSV-2
(G and Lyons) and against HSV-1 TK" (B2006) in embryonic skin muscle (E6SM) and human embryonic
lung (HEL) cell cultures. Ribavirin, ganciclovir, penciclovir, (S)-DHPA, BVDU and ACV were used as
reference compounds.
The cytostatic activity measurements were basically described [20]. Briefly, tumor cells were seeded
at 250,000-300,000 cells/ml in 200 tl-wells of 96-wells microtiter plates and incubated for 2 days (L1210,
FM3A) or 3 days (Molt4/CS, CEM) at 37C in a humidified CO2-controlled atmosphere. At the end of the
incubation period, cells were counted with a Coulter counter and the IC50 (50% inhibitory concentration)
determined as the compound concentration required to inhibit tumor cell proliferation by 50%.
59
H. Hadjiliadis et al.
A
c)
Synthesis, CharcterizJion andAntiviral
Protperties ofPd(H) Complexes with Penciclovir
6O
Metal BasedDrugs Vol. 8, Nr. 1, 2001
3.4 Broad-spectrum antiviral activity assays
For herpes simplex viruses (HSV), vaceinia virus (VV), Coxsackie virus type B4, vesicular stomatitis
virus (VSV), parainfluenza virus type 3, respiratory syncytial vires (RSV), Sindbis vires, Punta Toro virus and
reovirus type 1, the origin of the vires stocks [17] and the assay procedures [18, 19] have been described
previously. HSV assays were carried out against HSV-1 TK (KOS, F and Melntyre) and HSV-2 (G and Lyons)
and against HSV-1 TK- 032006) in embryonic skin muscle (F_SM) and human embryonic lung (HEL) cell
cultures. Ribavirin, gancielovir, peneiclovir, (S)-DHPA, BVDU and ACV were used as reference compounds.
The cytostatic activity measurements were basically described [20]. Briefly, tumor cells were seeded at
250,000-300,000 cells/ml in 200 pl-wells of 96-wells microtiter plates and incubated for 2 days (L1210,
FM3A) or 3 days (Molt4/C8, CEM) at 37C in a humidified CO2-controlled atmosphere. At the end of the
incubation period, calls were counted with a Coulter counter and the IC0 (50% inhibitory concentration)
determined as the compound concentration required to inhibit tumor cell proliferation by 50%.
TABLE IV. Cytotoxieity and antiviral activity of test compounds in HeLa cell cultures
Compound
cytotoxie
concentrationa Vesicular Coxsaekie
g/ml) stomatitis virus B4
virus virus
Minimum inhibitory concentrationb (,uegml)
Respiratory
syncytial
> 400 > 400 240 > 400
> 400 > 400 > 400 > 400
III > 400 > 400 > 400 > 400
IV > 400 > 400 > 400 > 400
V > 400 240 240 > 400
BVDU > 400 > 400 > 400 > 400
(_S)-DHPA > 400 240 > 400 > 400
Ribavirin >400 48 48 0.64
aRequired to cause a microscopically detectable alteration of normal cell morphology.
bRequired to reduce vires-induced eytopathogenieity by 50 %.
61
IV. Hadjiliadis et al. ynthesis, Characterization and.4ntiviral
Properties ofPd(II) Complexes with Penciclovir
A A A
'T
62
Metal Based Drugs Vol. 8, Nr. 1, 2001
TABLE VI. Inhibitory effects of test compounds on the proliferation of murine leukemia
cells (L1210/0), murine mammary carcinoma cells (FM3A) and human T-lymphocyte
cells (Molt4/C8, CEM/0)
Compound
Ill
L1210 FM3A
196 + 76 > 250
115 + 50 170 + 3
175 + 105 > 250
ICso
Molt4/C8 CEM
> 250 199 + 72
34 + 2 30 + 5
198 + 5 207 + 37
IV > 250 > 250 > 250 > 250
V 110 + 16 112 + 3 125 + 12 132 + 12
"50% inhibitory concentration.
4. References.
1. (a) G.B. Elion, P.A. Furman, J.A. Fyfe, P. de Miranda, L. Beauchamp, H.J. Schaeffer Proc. Natl. Acad. Sc.
USA. (1977), 74, 5717. (b) H.J. Schaeffer, L. Beauchamp, P. de Miranda, G.B. Elion, D.J. Bauer, P. Collins
Nature (London). (1978), 272, 583.
2. J.A. Fyfe, P. M. Keller, P. A. Furman, R. L. Miller, G. B. Elion, J. Biol. Chem., (1978) 253 8721
3. Shaw T., Amor P., Civitico G., Boyd M. and Locarnini S. Antimicrob. Agents Chemother, (1994) 38 719.
G. B. Elion, J. Antimicrob. Chemother., (1983) 12 Suppl. B 9
4. G.B. Elion, J. Antimicrob. Chemother., (1983) 12 Suppl. B 9
5. Williams R. J. P. Pure Appl. Chem. (1983) 55_ 35.
6. (a) L. Cavallo, R. Cini, J. Kobe, L.G. Marzilli, G Natile, J. Chem. Soc. Dalton Trans. (1991) 1867. (b) A.
Garcia-Raso, J. J. Fiol, F. Badenas, R. Cons, A. Terron, M. Quiros, J. Chem. Soc. Dalton Trans. (1999) 167.
7. I. Turel, I. Leban, K. Gruber, J. Inorg. Biochem. (1996) 63 41.
8. I. Turel, N. Bukovec, M. Goodgame, D. J. Williams, Polyhedron (1997) 16 1701.
9. (a) S. Grabner, J. Plavec, N. Bukovec, D. Di Leo, R. Cini, G. Natile, J. Chem. Soc. Dalton Trans. (1998)
1447.
10. Z. Balcarova, J. Kasparkova, A. Zakovska, O. Novakova, M.F. Sivo, G. Natile, V. Brabec, Mol. Pharm.
(1998) 53 846
11. J. Kobe, PATENT, International Application Number PCT/SI99/00021
12. G. Pneumatikakis, N. Hadjiliadis T. Theophanides, Inorg. Chem. 1978 17 915
13. N. Hadjiliadis, T. Theophanides, Inorg. Chim. Acta (1976) 16 77
14. N. S. Kurnakoff, J. Prakt. Chem. 1894 50 483.
15. N. Hadjiliadis, G. Pneumatikakis, J. Chem. Soc. Dalton Trans. 1978 1691
16. K. Nakamoto, "Infrared and Raman Spectra ofInorganic and Coordination Compounds", 5th
Ed. J. Wiley
and Sons, Inc. 1997, Part B, p. 185.
17. M. Witvrouw, D. Daelemans, C. Pannecouque, J. Neyts, G. Andrei, R. Snoeck, A.-M. Vandamme, J.
Balzarini, J. Desmyter, M. Baba, E. De Clercq, Antiviral Chem. Chemother. (1998) 9 403-411.
18. E. De Clercq, M. Luczak, J.C. Reepmeyer, K.L. Kirk, L.A. Cohen, Life Sci. (1975) 17 187-194.
19. E. De Clercq, J. Descamps, G. Verhelst, R.T. Walker, A.S. Jones, P.F. Torrence, D. Shugar, J. Infect. Dis.
(1980) 141 563-574.
J. Balzarini, E. De Clercq, M.P. Mertes, D. Shugar, P.F. Torrence, Biochem. Pharmacol. (1982) 31, 3673-3682.
63

				

